# Otx but Not Mitf Transcription Factors Are Required for Zebrafish Retinal Pigment Epithelium Development

**DOI:** 10.1371/journal.pone.0049357

**Published:** 2012-11-05

**Authors:** Brandon M. Lane, James A. Lister

**Affiliations:** Department of Human and Molecular Genetics, Virginia Commonwealth University School of Medicine, Richmond, Virginia, United States of America; Purdue University, United States of America

## Abstract

Otx and Mitf transcription factors have been implicated in the development of the retinal pigmented epithelium (RPE), but the relationship between these factors and their specific roles in the development of the RPE have not been fully defined. The role of the three Otx transcription factors (Otx1a, Otx1b, and Otx2) and two Mitf transcription factors (Mitfa and Mitfb) in the development of the zebrafish RPE was explored in these experiments. The loss of Otx activity through morpholino knockdown produced variable eye defects, ranging from delayed RPE pigmentation to severe coloboma, depending on the combination of Otx factors that were targeted. Expression analysis through in situ hybridization demonstrates that *otx* transcription factors are necessary for the proper expression of *mitfa* and *mitfb* while Mitf transcription factors are not required for the expression of *otx* genes. Surprisingly, the loss of Mitf activity in *mitfa*, *mitfb*, or double *mitf* mutant zebrafish had no effect on RPE pigmentation or development. Moreover, histological analysis revealed that retinal lamination is unaffected in *mitf* mutants, as well as in *otx* morphants, even in regions lacking RPE. Otx and Mitf combined loss of function experiments suggest that *mitfa* and *mitfb* may still influence zebrafish RPE development. This is further supported by the ability of *mitfa* to induce pigmentation in the zebrafish retina when misexpressed. These findings suggest that one or more Otx targets in addition to *mitfa* and *mitfb*, possibly another *mitf* family member, are necessary for development of the RPE in zebrafish.

## Introduction

The retinal pigmented epithelium (RPE) is a monolayer of cells that lies between the retina and choroid and serves a variety of functions in the developing and mature eye [1]. These include providing nutritional support to the retina, regulating oxidative stress, and maintaining the outer segments of the photoreceptors. Defects in the RPE are implicated in a number of vision-related disorders such as age-related macular degeneration and retinitis pigmentosa [2], [3]. The RPE also plays a vital role in early eye development. During the formation of the optic cup, the RPE encompasses the retina and completes the formation of the cup with the anterior and posterior sides of the presumptive optic cup joining together at the ventral optic fissure [4]. Failure of the RPE to develop properly at the optic fissure can lead to a gap in the ocular layers known as a coloboma, and in more severe instances can lead to retinal degeneration resulting in microphthalmia or anopthalmia [5]. Microphthalmia and the more severe anophthalmia are diagnosed in up to 25% of children with severe vision impairments and in approximately 1 of every 3,500 live births [6]. The zebrafish presents an excellent model to study these ocular disorders due to the similarities between human and zebrafish eye development, cell composition, and morphology [7].

Unlike the pigmented cells found in the skin and hair, which develop from migrating neural crest cells, the RPE develops from the neuroepithelium of the optic vesicle. All naïve cells of the developing optic vesicle originally express a similar set of transcription factors and can be induced to adopt either a RPE or retinal fate [8], [9]. A combination of signals from surrounding tissues can induce the differentiation of these two cell types through the activation of retinal or RPE-specific transcription factors [10]. Two transcription factors that are up-regulated in the prospective RPE and are required for proper RPE differentiation in several species are Otx2 and Mitf [11], [12].

Orthodenticle-related (Otx) proteins are paired type homeobox transcription factors that serve an essential role in vertebrate anterior head and brain development. In mice, Otx2 is initially expressed in the entire developing optic vesicle and is later restricted to the differentiating RPE [13]. Complete loss of *Otx2* function in mice results in embryonic lethality, while heterozygous mutations have been found to produce RPE phenotypes, particularly when present in an *Otx1* null background [12]. The ocular phenotype of *Otx* mutants is highly variable and is characterized by degradation of the retinal layers and a second retina-like unpigmented layer of cells replacing the RPE [12], [14]. Heterozygous mutations in humans have also been identified with a similar ocular phenotype that varies from a small coloboma to a complete loss of ocular tissue [15].

Zebrafish, like other teleosts, underwent a genome duplication during evolution that resulted in many cases in two copies of genes present as single copies in mammalian genomes [16], [17]. Zebrafish possess three orthologs, *otx1a*, *otx1b*, and *otx2*, to the two mammalian *Otx1* and *Otx2* genes. Zebrafish Otx1a and Otx2 are evolutionarily well conserved compared to murine proteins with a 78% and 94% amino acid identity respectively [18], [19]. The expression patterns and knockdown phenotypes of Otx1a and Otx2 suggest that these proteins serve conserved and partially redundant functions in zebrafish anterior brain and eye development. [20]. A third zebrafish *otx* gene, *otx1b*, encodes a protein with almost equal similarities in amino acid composition to both murine Otx1 and Otx2, and a similar expression pattern to *otx1a* and *otx2* [18].

Microphthalmia-associated transcription factor (Mitf) is a member of the basic helix-loop-helix/leucine zipper family of transcription factors. The mammalian *Mitf* gene produces numerous isoforms through the use of at least nine different alternative promoters and alternative splicing [21]. Mutations in mice that affect common exons, or RPE-specific isoforms of *Mitf* can result in the microphthalmia phenotype for which the transcription factor is named. The reduction in eye size is due to incomplete closure of the optic fissure and retinal degeneration, leading to a phenotype that can vary from a simple coloboma to complete anopthalmia [22], [23]. The degree of severity can be extremely variable not only between animals with the same mutation, but also between eyes of the same animal [22]. A common feature of the phenotype includes the transdifferentiation of dorsal RPE into an additional hyperproliferative retinal layer.

The first *mitf* gene identified in zebrafish was *mitfa*, which shares the most similarity with the mouse neural crest specific *MitfM* isoform [24]. However, *mitfa* is expressed in the RPE as well as neural crest cells of the developing zebrafish embryo. Despite its expression pattern, null mutations in the *mitfa* gene affect only the neural crest-derived cells, leaving the RPE unaffected. A further examination of the zebrafish genome revealed a second *mitf* gene, *mitfb*, expressed in the developing RPE but not neural crest melanocytes and encoding a protein with greater similarity, especially in the amino terminal region, to Mitf isoforms present in the mouse RPE [25]. These findings suggested that the RPE and neural crest functions of the single mammalian gene may be partitioned between the duplicate zebrafish *mitf* genes.

In addition to their individual roles in RPE development, evidence suggests a complex relationship between Otx and Mitf transcription factors. In vitro experiments have revealed that Otx2 and Mitf have the ability to form protein complexes and that maximal target gene expression is achieved when both proteins are present [14], [26]. Analysis of murine *Otx2* and *Mitf* mutants has led a model involving positive feedback regulation between the two factors [14]. Analysis of mouse models has revealed that *Otx2* expression is eliminated in *Mitf* mutants and that *Mitf* expression is severely down-regulated in *Otx2* heterozygous mutants [10], [12]. This feedback relationship was further supported by findings in conditional *β-catenin* knockouts with a RPE deficient phenotype, as both *Otx2* and *Mitf* were present in patches of pigmented RPE cells but absent in the unpigmented regions [27].

In the experiments described here, we show that knockdown of Otx activity creates ventral RPE deficits and coloboma without an effect on retinal lamination. Otx1a and Otx2 appear to play the primary roles in RPE development, with Otx1b having a smaller contribution. In contrast, the Mitf transcription factors Mitfa and Mitfb are not required for zebrafish RPE development, but the combined knockdown of Mitf and Otx activity suggests that Mitf factors may still be involved in RPE development, and moreover Mitfa is capable of inducing pigmentation upon misexpression in the developing retina. Together these data indicate that the Otx transcription factors and targets other than, but possibly including, the Mitfs are necessary for development of the RPE and proper eye morphogenesis in zebrafish.

## Materials and Methods

### Ethics statement

All zebrafish procedures were performed in compliance with protocol AM10125, approved by the Institutional Animal Care and Use Committee of Virginia Commonwealth University.

### Zebrafish lines

Wildtype embryos were obtained through natural matings of adults of the AB strain or AB/WIK hybrids and were staged according to Kimmel et al. [28]. Genotyping of *mitfa^w2^* and *mitfa^b692^* fish lines was performed as described in [24]. *mitfb* mutant alleles *hu3857* and *hu3561* were obtained from the ZF-MODELS consortium. Genomic DNA from each allele was sequenced to confirm the mutations. Genotyping of each line was achieved through PCR with the primers listed in Table 1, followed by restriction enzyme digestion: the nucleotide change in *hu3561* creates an AlwNI site while *hu3857* destroys an MboI site.

**Table 1:** Oligonucleotides used in this study.

Name	Sequence
hu3561F	5′ AGC ATA ATA GGT CCC CTT TAA C 3′
hu3561R	5′ AAT ACA TGT AAA ACC TGA AAA GC 3′
hu3857F	5′ AGC GCC CCC AAC AGT CCC AGG GCC T 3′
hu3857R	5′ CTG TGG CGA CCC CGG ATT AAT AAA GGG AC 3′
otx1aF	5′ AAG GGC CTT AAA TAT CTC TGC 3′
otx1aR	5′ GGA TCC ATT AAC CCT CAC TAA AGG GAA CAC ACT TTG GTA AGC TCT GG 3′
otx2F	5′ ACC ATG ATG TCG TAT CTC AAG CAA C 3′
otx2R	5′ TCA CAA CAC TTG GAA TTT CCA GGA 3′
silvbF	5′ TGG ATA ACC GTA TTA CCG CC 3′
silvbR	5′ CGC GCA ATT AAC CCT CAC TAA AGC ACT AGT CAT ACC AGG ATC 3′

The *ET(-1.5hsp701:Gal4-VP16)s1003t;Tg(UAS-E1b:-Kaede)s1999t* double transgenic line was acquired from the Zebrafish International Resource Center (ZIRC). Adult transgenic fish were outcrossed to AB/WIK wild-type fish and embryos with no Kaede fluorescence were selected, raised, and genotyped at three months' of age to determine *s1003t* (*Gal4-VP16*) carrier status. The UAS:mitfa construct was created using the Tol2 kit [29]. Briefly, p3E-2A-FLAG-mitfa-pA was combined with p5E-UAS-TP2, pME-GFP-nostop, and pDestTol2cryaa-DsRedpA to create pDestTol2cryaa-DsRedpA-UAS-TP2/EGFP-nostop/2A-FLAG-mitfa-pA. The UAS vector was combined in a 1∶1 ratio with transposase mRNA for a final concentration of 25 ng/µl and injected into one to two cell wildtype AB embryos. Embryos with DsRed expression in the lens at 52 hpf were selected and raised to maturity and then mated pair-wise with wildtype AB/WIK fish. Putative germline founders (i.e., producing embryos with red lenses) were then mated directly to *s1003t* carriers.

### Morpholino knockdown

Previously validated translation-blocking morpholino oligonucleotides against *otx1a* and *otx2* [20], and against *otx1b* [30] were obtained from Gene Tools (Philomath, OR). A morpholino designed against the 5′ leader sequence found in the pCS2 expression vector, with sequence 5′ GAT CCT GCA AAA AGA ACA AGT AGC T 3′, was used as a control in all experiments. All morpholinos were injected at a concentration of 1.25 ng/embryo each unless otherwise noted. Larvae at five days post fertilization (dpf) were anesthetized in Tricaine and each eye was examined using a SZX12 dissecting stereomicroscope with DP70 camera (Olympus) and scored. To reduce the variability associated with morpholino injections when *otx* and *mitf* interactions were examined, larvae were only scored for phenotype when embryos of all four *mitf* genotypes (i.e. wild-type, both single mutants, and the double mutant) were successfully injected with the same needle. This experiment was repeated until at least three trials were obtained for each morpholino/morpholino combination. *mitfa^w2^* was used as the representative *mitfa* single mutant in all experiments.

### Histology

Specimens for histology were fixed in 4% PFA, equilibrated in 30% sucrose in PBS, and embedded in Tissue-Tek O.C.T (Sakura Finetek, Torrance, CA) prior to sectioning on a Shandon cryostat microtome (GMI, Ramsey, MN). 12 micron sections were stained in 1% Methylene blue, mounted in PBS and photographed with a Spot RT CCD camera (Diagnostic Instruments) on a Nikon ECLIPSE E800M microscope at 20× zoom. Measurements of the images were made using ImageJ software. Images were processed using Adobe Photoshop and Helicon Focus software.

### In-situ hybridization

Plasmid templates for synthesis of digoxigenin-labeled RNA probes were generated by PCR using the primers listed in Table 1; *otx1a* was amplified from IMAGE clone 7138889 and cloned into pCR2.1-TOPO (Invitrogen), *otx2* (NM 1311251, nt 264–1133) was amplified from 24 hpf whole embryo first-strand cDNA and cloned into pCRII-TOPO (Invitrogen). The *otx1a* template was linearized with XhoI and transcribed with T3 RNA polymerase; while that for *otx2* was linearized with Xhol and transcribed with SP6 RNA polymerase. *silvb* was amplified from IMAGE clone 6891818 and the product used directly as a template for transcription with T3 RNA polymerase. Probes for *dct*, *mitfa* and *mitfb* have been described previously [24], [25], [31]. In situ hybridization was carried out as described previously [32].

### Immunohistochemistry

Larvae were anesthetized in Tricaine at 5 dpf and fixed in 4% PFA overnight. Immunohistochemistry was performed as described in Macdonald [33] except for the permeabilization step which was performed with proteinase K. The Zpr1 monoclonal antibody ([34], obtained from ZIRC) and Alexa 488 goat anti-mouse secondary antibody (Invitrogen) were each used at a dilution of 1∶1000. After staining, larvae were sectioned at 25 microns and covered with vectashield (Vector laboratories) for imaging on a Leica TCS SP2 AOBS inverted laser scanning confocal microscope.

### Statistics

For comparison, at least seven sections from each genotype were averaged for the measurements of RPE thickness, outer nuclear layer thickness and total or dorsal eye length. ANOVA analysis was performed using the online calculator at http://www.physics.csbsju.edu/stats/anova.html. Combined *otx* and *mitf* knockdown/mutant data were first analyzed for significant interactions between the genotype and trials based on phenotype using R software. There was a significant interaction in the *otx1a*/*otx2* combination morpholino experiments and therefore these had to be analyzed on a trial by trial basis. Further analysis was performed through the use of Fisher's exact T test.

## Results

### Otx expression is required for proper zebrafish RPE development

**Figure 1:**
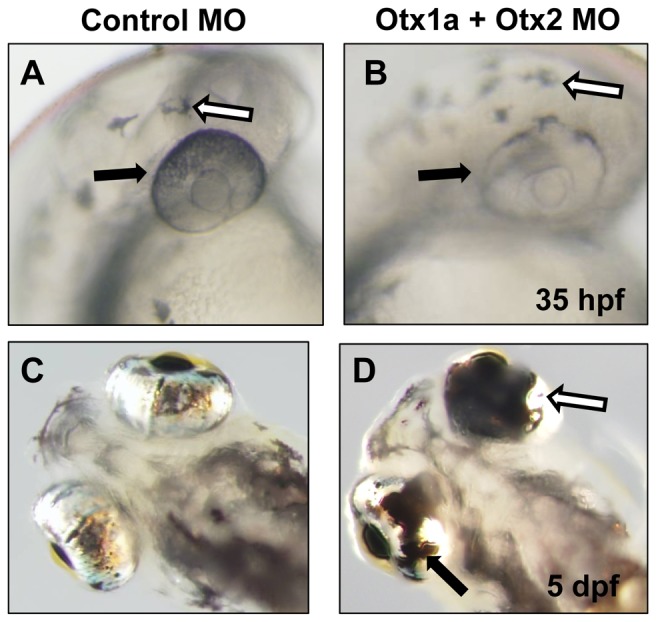
Otx morpholino knockdown results in a variable degree of RPE deficits in zebrafish. (A–B) Control morpholino (A) and *otx1a*/*otx2* morpholino (B) injected embryos photographed at 35 hpf. RPE pigmentation is reduced in *otx1a*/*otx2* morphants (black arrows) while neural crest derived melanocytes are unaffected (white arrows). (C–D) Ventral view of 5 dpf control morpholino (C) and *otx1a*/*otx2* morpholino (D) injected larvae. The phenotype of *otx1a*/*otx2* morphants can vary in severity between eyes of the same embryo, with one eye displaying a minor coloboma phenotype (black arrow) and the other eye displaying a major coloboma phenotype (white arrow).

Previous analysis of zebrafish *Otx* genes suggested that in addition to anterior brain defects, knockdown of Otx transcription factors can result in eye abnormalities [20]. We investigated the effect of the individual and combined loss of function for Otx transcription factors using previously characterized translation-blocking morpholinos for *otx1a* and *otx2* [20] and for *otx1b* at a concentration of 1.25 ng per embryo for each morpholino [30]. Knockdown of any of the Otx factors singly did not result in noticeable deficits in RPE pigmentation. The combination of *otx1a*/*otx1b* or *otx1b*/*otx2* morpholinos did produce the occasional absence of ventral RPE pigmentation but the combination of *otx1a* and *otx2* morpholinos consistently produced a significant delay in RPE pigmentation, leaving the neural crest derived melanocytes unaffected (Fig.1A,B). RPE pigmentation began to recover around 72 hpf, possibly due to the transient nature of the knockdown produced by the morpholinos.

**Figure 2:**
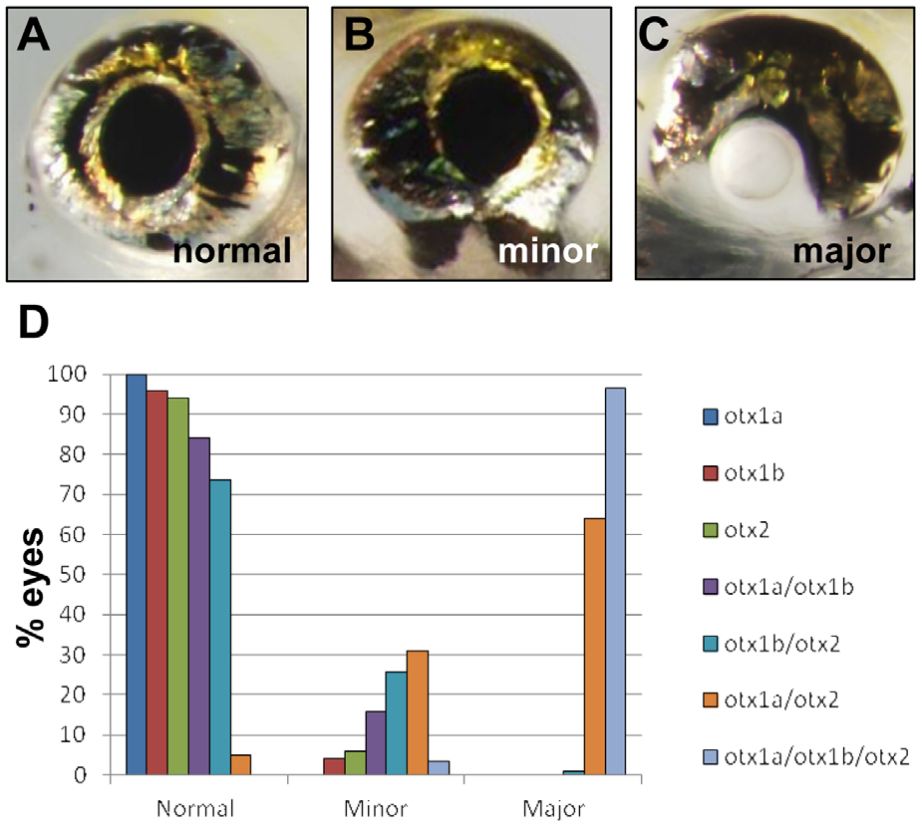
Otx morphant phenotypes. (A–C) Eye phenotypes, assessed at 5 dpf, were scored as normal when the RPE displayed no visible defects (A), minor when coloboma was present but anterior and posterior ventral RPE were still contiguous (B), and major when the anterior and posterior ventral RPE were completely separated (C). (D) Histogram showing the eye defects induced by *otx* gene knockdown when injected at a morpholino concentration of 1.25 ng/embryo. Knockdown of individual *otx* genes caused only minor defects, while knockdown of *otx1a* and *otx2* together, or all three *otx* genes together, produced a majority of embryos with major eye defects. *otx1a*, N = 108; *otx1b*, N = 230; *otx2*, N = 122; *otx1a/otx1b*, N = 222; *otx1b/otx2*, N = 256; *otx1a/otx2*, N = 214; *otx1a/otx1b/otx2*, N = 178.

At 5 dpf, the *otx* morphant phenotype was extremely variable both between larvae and between eyes of a single fish (Fig 1C,D). To quantify the effect, each eye from 5 dpf larvae was examined and scored as having either a normal RPE (no discernible phenotype), mild RPE deficits (incomplete closure of the optic fissure with a coloboma, but still an anterior and posterior connection ventrally), or major RPE deficits (complete lack of ventral RPE with no connection between the anterior and posterior RPE) (Fig. 2A–C). Similar to what is observed in murine *Otx* mutants and in the zebrafish pigmentation phenotypes at 35 hpf, the knockdown of *otx1a*, *otx1b*, or *otx2* had little effect on eye phenotype (0%, 3% and 6% minor phenotype respectively) (Fig. 2D). The combination of *otx1a*/*otx1b* and *otx1b*/*otx2* morpholinos resulted in a higher percentage of minor phenotypes but failed to increase the severity of the eye phenotype. The combination of *otx1a* and *otx2* morpholinos however, resulted in defects in nearly all larvae (95%) with most (64%) having major disruptions of the developing RPE. These results suggest that Otx1a and Otx2 have the major, though partially redundant roles in zebrafish RPE development. The combination of *otx1b* with either *otx1a* and *otx2* morpholinos does not greatly increase the severity of the RPE phenotype, but the elimination of all three *otx* factors creates a significantly more severe phenotype than what is observed in *otx1a*/*otx2* morphants. This increase in severity was consistent even when the total concentration of the triple otx morpholino combination was reduced to that of the *otx1a*/*otx2* morpholino combination (data not shown).

**Figure 3:**
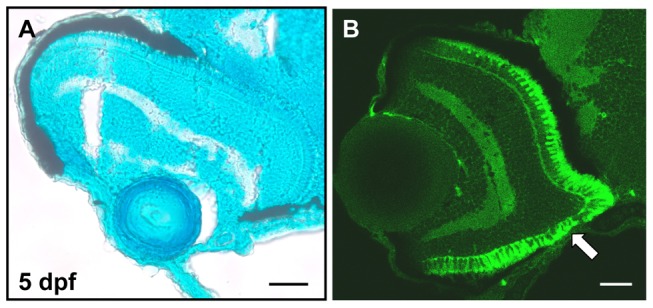
The loss of ventral RPE in *otx* morphants has little effect on retinal lamination. (A) Methylene blue stained sagittal section of an *otx1a*/*otx2* morphant classified with a minor eye phenotype. The laminar structure of the retina is unaffected despite extensive absence of RPE. (B) Zpr1 staining reveals that red-green double cone photoreceptors differentiate normally despite the loss of RPE (white arrow). Scale bar is 25 micrometers.

Any loss of RPE in zebrafish *otx* morphants occurs at the proximal ventral point of the optic cup and RPE deficits extend distally and dorsally with more severe phenotypes. Minor loss of RPE in these morphants does not have any noticeable effect on retinal lamination, although some layers may be thinner (Fig. 3A). Major loss of RPE can disrupt the eye cup shape in the ventral portion of the eye with a variable loss of ventral ocular tissue. A rotation of the entire eye up to 90 degrees is also observed in many of the more severe RPE deficient zebrafish larvae (data not shown). Despite the rotation and missing tissue, the retina appears to be well organized in areas of the eye lacking RPE. *otx* morphants also do not display any microphthalmia except for the occasional loss of ventral tissue. The dimensions of the ocular layers in the dorsal region of the eye remain comparable to wildtype eyes, even in the remaining RPE immediately adjacent to the affected ventral area (Figure S1).

The specific loss of ventral RPE in zebrafish *otx* morphants is similar to what has been described in murine *Otx* mutants. However in zebrafish *otx* morphants, the affected RPE does not appear to transdifferentiate into the retinal–like cells observed in the mouse and instead is merely absent (Fig. 3A). In addition to examining retinal lamination, 5 dpf larvae were assessed using the monoclonal Zpr1 antibody [34) that is specific for red/green double cone photoreceptors (Fig.3B). No additional layer of cone photoreceptors was observed in RPE deficient larvae and the photoreceptors appear largely unaffected by the loss of RPE in *otx* morphants.

**Figure 4:**
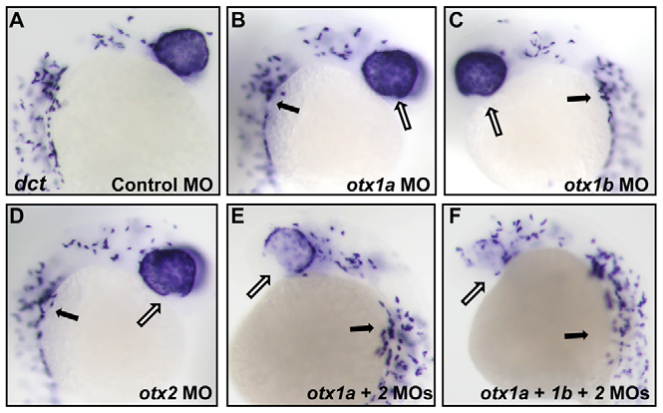
*Otx* knockdown causes a decrease in *dct* expression in the developing RPE. *dopachrome tautomerase* (*dct*) mRNA expression was analyzed through in situ hybridization at 24 hpf in *otx* morphants. *dct* expression was reduced only in the ventral most region of the optic cup in single morphants (B–D, white arrows) compared to controls (A). *dct* expression was significantly decreased in the ventral RPE of *otx1a*/*otx2* morphants and to an even greater degree in *otx1a*/*otx1b*/*otx2* morphants (E–F, white arrows). *dct* expression in neural crest melanocytes was unaffected (black arrows). Approximately 50 larvae were examined for each condition.

Consistent with what is observed in the RPE phenotype and pigmentation analysis, the injection of any of the three morpholinos alone rarely produced small ventral deficits in the expression of melanin biosynthesis pathway gene *dopachrome tautomerase* (*dct*) when examined through in situ hybridization at 24 hpf, but the combination of *otx1a* and *otx2* morpholinos created a significant loss of *dct* expression in the ventral RPE (Fig. 4). Likewise, analysis of the RPE specific *silvb* gene [35] at 24 hpf revealed a small area of absent expression in the ventral portion of the eye in a small percentage of single *otx1a*, *otx1b*, or *otx2* morphants, and greater deficits resulting from the combination of *otx1a*/*otx2* morpholinos and in *otx* triple morphants (Figure S2).

### Otx expression positively regulates the expression of mitfa and mitfb

**Figure 5:**
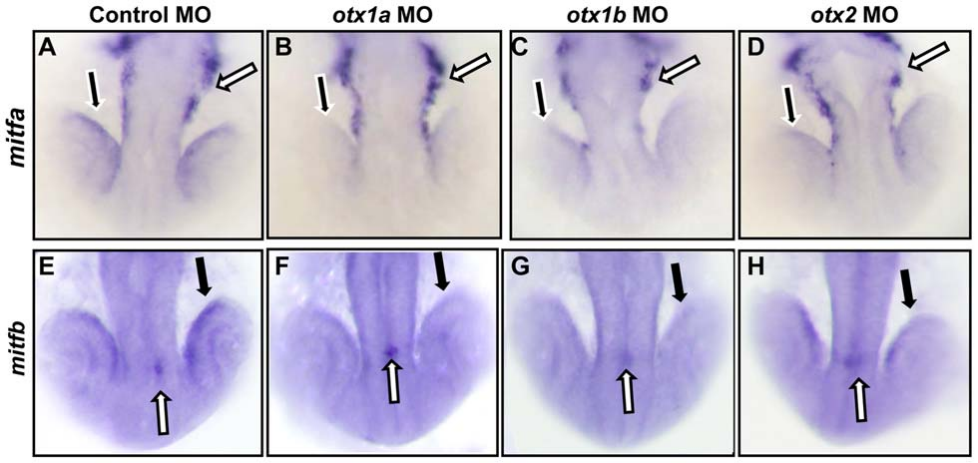
*mitfa* and *mitfb* expression is decreased in *otx* morphants. Expression of *mitfa* (A–D) and *mitfb* (E–H) was examined through in situ hybridization at the 21 somite stage in embryos injected with a control morpholino (A,E), or morpholinos against *otx1a* (B,F), *otx1b* (C,G), or *otx2* (D,H). In situ hybridization analysis revealed a decrease in *mitfa* expression in the developing RPE cells (black arrows) but not in the neural crest cells (white arrows) of *otx* single morphants (D) and when compared to controls (A). *mitfb* expression was also reduced specifically in the RPE (black arrows) of *otx* morphants (F–H) compared to the controls, leaving epiphysis expression unaffected (white arrows). All morphants were processed simultaneously with approximately 50 larvae for each condition and the experiment was repeated twice.

In murine experiments analyzing the regulatory relationship between Otx and Mitf transcription factors, the presence of either factor was found to be dependent on the other [10], [12]. We therefore examined *mitfa* and *mitfb* expression through in situ hybridization at 21hpf in *otx1a*, *otx1b*, and *otx2* single morphants as well as in *otx1a*/*otx2* morphants. The expression of *mitfa* in the RPE was reduced by all three *otx* single morpholino knockdowns while the expression in the neural crest remained unaffected (Fig. 5A–D). Expression of *mitfb* in the RPE but not in the epiphysis was also significantly decreased in *otx1a*, *otx1b*, and *otx2* morphants (Fig. 5E–H). The reduction in *mitfa* and *mitfb* expression varied somewhat between embryos, but *otx1a* morphants consistently displayed a greater reduction in expression when compared to *otx1b* and *otx2* morphants (data not shown).

### Mitfa and Mitfb are not required for zebrafish RPE development

**Figure 6:**
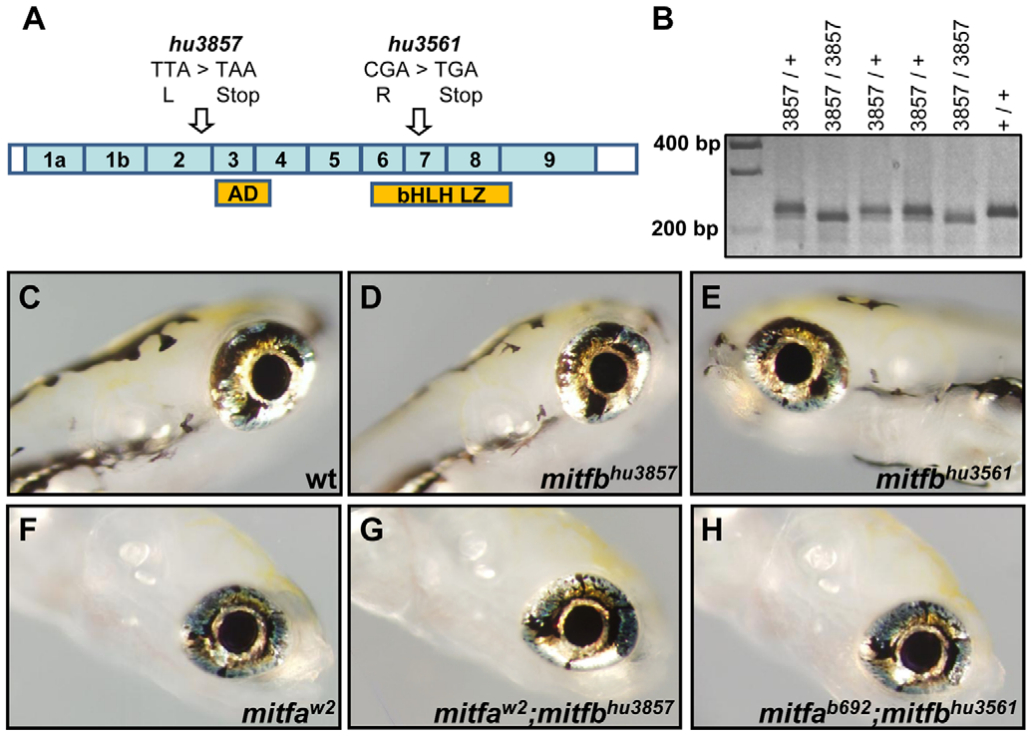
Elimination of *mitfa* and *mitfb* activity has no detectable effect on RPE development in zebrafish embryos. (A) Diagram of *mitfb* cDNA showing exons, functional domains, and locations of mutations (arrows). The *mitfb^hu3857^* allele contains a T>A nonsense mutation that causes a switch from leucine to a stop codon in exon 2. The *mitfb^hu3561^* allele has a C>T nonsense mutation that results in a switch from Arginine to a stop codon in exon 7, in the basic-helix-loop-helix/leucine zipper binding domain (bHLH LZ). AD, transcriptional activation domain. (B) The *mitfb^hu3857^* allele creates an AlwNI restriction site, allowing genotyping of animals by electrophoresis of digested PCR products. (C–H) Bright field images depicting the normal RPE pigmentation and eye development in 5 dpf larvae; wildtype (C), or *mitfb^hu3857^* (D), *mitfb^hu3561^* (E), *mitfa^w2^* (F), *mitfb^hu3857^*;*mitfa^w2^* (G), or *mitfb^hu3561^*;*mitfa^b692^* (H) homozygous mutants.

The loss of *mitfa* in zebrafish is not associated with eye defects, however *mitfa* is co-expressed with a second *mitf* gene, *mitfb*, at early stages [25]. To examine *mitfb* function in the developing RPE two mutant alleles were obtained (Fig. 6A). The *mitfb^hu3561^* allele has a nonsense mutation in exon 7 causing an amino acid change from arginine to a stop codon, which disrupts the putative binding domain of the protein. The *mitfb^hu3857^* mutant allele has a nonsense mutation in exon 2 causing a switch from leucine to a stop codon. Each of the two *mitfb* mutant alleles were intercrossed, however no obvious abnormalities in homozygous *mitfb* embryos or larvae were observed (Fig. 6C–E). 5 dpf homozygous mutant larvae were cryosectioned, stained with methylene blue, and photographed. When the dimensions of the eye and the thickness of several ocular layers were examined, the analysis revealed no significant differences between the *mitfb* mutants and age-matched wildtype larvae in RPE thickness, outer nuclear layer thickness, or overall eye length (Fig. S3).

Because the possibility existed that *mitfa* was able to compensate for the loss of Mitf activity in *mitfb* mutants [25], *mitfa* null alleles *mitfa^b692^* and *mitfa^w2^* were crossed to the *mitfb^hu3561^* and *mitfb^hu3857^* alleles respectively and then bred to obtain homozygous *mitfa*;*mitfb* double mutant embryos. However, these *mitfa*;*mitfb* double mutants also did not display any RPE or ocular phenotype when compared to either age-matched wildtype or *mitfa* mutant embryos (Fig. 6F–H). There were no changes in photoreceptor layer for abnormalities at 5 dpf using the Zpr1 monoclonal antibody in either set of *mitfa*;*mitfb* double mutants when compared to wildtype or *mitfa* and *mitfb* single mutants (data not shown). In addition to not being necessary to early development, *mitfa* and *mitfb* do not appear to be required for the maintenance of the RPE layer during later life, as sectioned eyes of adult *mitf* single and double mutants, which are viable, do not show any significant changes in the RPE or retinal layers (data not shown).

### Mitf transcription factors may still have role in RPE development

**Figure 7:**
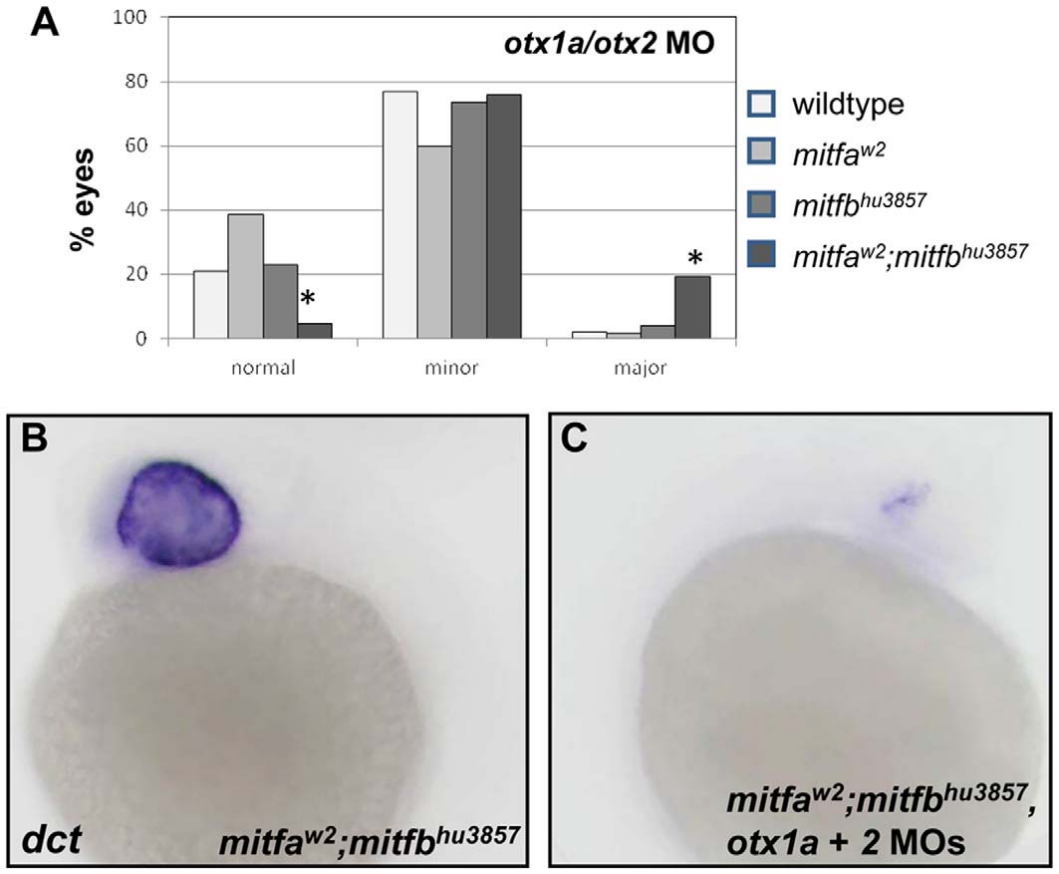
The combined knockdown of Otx and Mitf transcription factors creates a more severe phenotype. (A) The effect of *otx1a*/*otx2* knockdown was compared between wildtype, *mitfa^w2^* and *mitfb^hu3857^* single mutants, and *mitfb^hu3857^*;*mitfa^w2^* double mutants. Morpholinos were injected at a total concentration of 2 ng/embryo using the same needle for all genotypes. Results from one representative experiment are shown. The percentage of severe RPE defects was significantly increased (P<0.0001) in the *mitfb^hu3857^*;*mitfa^w2^* double mutant background compared to wildtype and single *mitfa* and *mitfb* mutants. Wildtype, N = 252; *mitfa*, N = 196; *mitfb*, N = 158; *mitfa*;*mitfb*, N = 170. (B,C) At 24 hpf *dct* expression is nearly eliminated in *mitfb^hu3857^*;*mitfa^w2^* embryos injected with the *otx1a*/*otx2* morpholino combination (C) compared to sibling embryos injected with control morpholino (B).

The combined knockdown of Otx and Mitf transcription factors was explored to determine if *mitfa* and *mitfb* may still contribute to RPE development. To accomplish this, *otx1a* and *otx2* morpholinos were injected into wildtype, *mitfa* mutant, *mitfb* mutant, and *mitfa*;*mitfb* double mutant embryos to determine if the loss of Mitf activity would increase the severity of the *otx* morphant phenotype. Phenotypic variability of the combination of *otx1a*/*otx2* morpholinos, even at reduced concentration, precluded the analysis of multiple trials as a single group, but each trial on its own had a significant increase in the percentage of *mitfa*;*mitfb* double mutant embryos with major RPE deficit phenotypes when compared to wildtype and single *mitf* mutant embryos (Fig. 7A and Fig. S4). In addition to the increased phenotypic severity, the combined knockdown of *Otx1a* and *Otx2* also produced a significant decrease in *dct* expression in *mitfa*;*mitfb* double mutants when compared to wildtype *otx* morphants and uninjected *mitf* mutants (Fig. 7B,C; compare to Fig. 4E). Injections of either *otx1a* or *otx2* morpholinos singly into *mitfb^hu3857^* mutants produced a significant increase in both the percentage of embryos with a phenotype, as well as the severity of those phenotypes in *mitfa*;*mitfb* double mutants when compared to the other genotypes (Fig S5). Taken together, this evidence suggests that *mitfa* and *mitfb* interact with *otx* to contribute to zebrafish RPE development although they are not necessary on their own.

**Figure 8:**
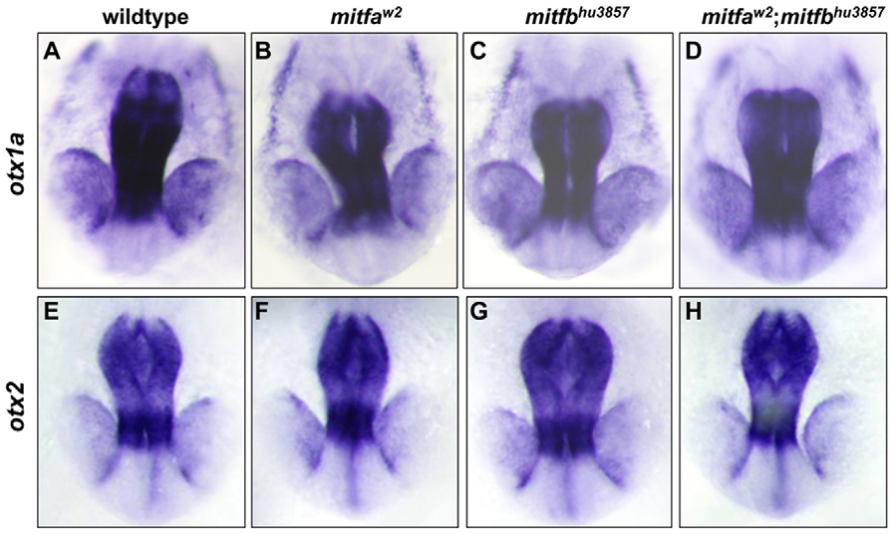
*otx1a* and *otx2* expression is not altered in *mitfa* and *mitfb* mutants. (A–D) No difference in *otx1a* expression was revealed through in situ hybridization in wildtype (A), *mitfa^w2^* (B), *mitfb^hu3857^* (C), and *mitfb^hu3857^*;*mitfa^w2^* (D) embryos at 21 hpf. (E–H) Likewise, *otx2* expression was unchanged in wildtype (E), *mitfa^w2^* (F), *mitfb^hu3857^* (G), and *mitfb^hu3857^*;*mitfa^w2^* (H) embryos at the same stage. All mutants were processed simultaneously with approximately 50 larvae examined for each condition and the entire experiment was repeated.

The expression of *otx1a* and *otx2* was also examined through in situ hybridization at 21hpf in *mitfa^w2^* and *mitfb^hu3857^* single and double mutants. There was no observable change in *otx1a* or *otx2* expression in any of the *mitfa^w2^* and *mitfb^hu3857^* single or double homozygous mutants when compared to wildtype embryos (Fig. 8). This unidirectional regulation of Otx transcription factors differs from what is observed in mice and may help to explain why loss of Mitf transcription factors does not have an apparent effect on zebrafish RPE development.

### Mitfa has the ability to promote pigmented cell fate in the developing retina

**Figure 9:**
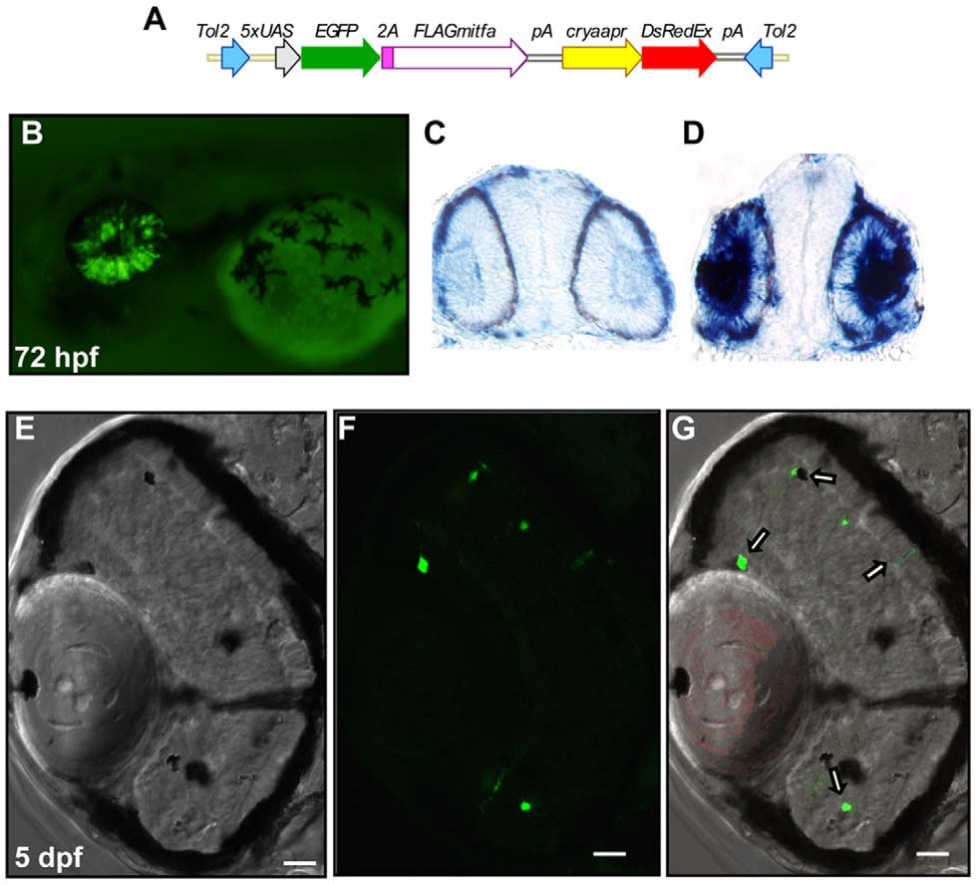
The misexpression of *mitfa* in the developing retina is capable of inducing foci of pigmentation. (A) Schematic of the Tol2 transposon construct expressing EGFP and FLAG-tagged Mitfa, linked by a viral 2A peptide, from a 5×UAS promoter, and containing an *alpha crystallin* promoter:DsRed-Express transgene marker. (B) 72 hpf larva doubly transgenic for *s1003t* (retinal Gal4-VP16) and *UAS:EGFP-2A-FLAG-mitfa*, expressing GFP in the developing retina. (C,D) 20 micron cryosections of 28 hpf larvae examined for *mitfa* expression through in situ hybridization revealed *mitfa* expression in the developing retina and lens of a GFP expressing embryo (D) but not in an embryo lacking GFP expression (C). (E–G) cryosections of a 5 dpf *s1003t*; *UAS:EGFP-2A-FLAG-mitfa* transgenic larva, displaying foci of pigmentation (E) and GFP expression (F) in the retina. (G) Overlay demonstrating extensive overlap of GFP expression and ectopic pigmentation (white arrows). Scale bar indicates 20 micrometers in (E–G). Expression of DsRed-Express is also visible in the lens in (G).

Due to the similar developmental origin, RPE and retinal cells have the ability to transdifferentiate between each cell type for an extended period of time in many organisms [36]–[38]. Loss of Mitf activity in murine and quail mutants causes hyperproliferation and a conversion of the prospective RPE layer into a second inverted retinal layer [10], [39], [40]. Conversely, the retinal misexpression of *Mitf* is able to direct the transdifferentiation of retinal cells into a second pigmented layer [22], [41], [42]. To examine the ability of Mitfa to promote an RPE cell fate in developing retinal cells, a transgenic line was created with a 5× UAS sequence driving expression of *mitfa* in tandem with an EGFP reporter, along with an *alpha crystallin* promoter driving expression of DsRed in the developing lens to allow for identification of germline transformants (Fig. 9A).

*UAS:mitfa* founders were bred to the enhancer trap line *Et(-1.5hsp701:Gal4-VP16)s1003t*, expressing Gal4-VP16 in the developing retina [43]. A portion of embryos from such matings were observed to have GFP expression in the developing retina starting around 24 hpf, with robust expression by 28 hpf (Fig 9B). GFP expression was not observed in embryos that did not also later express DsRed in the developing lens. A small percentage of embryos also displayed scattered GFP expression and pigmentation in other tissues including the heart, where this expression was often accompanied by circulation problems (data not shown). When sorted for GFP expression and processed for in situ hybridization with a probe for *mitfa* at 28 hpf, only GFP expressing embryos displayed *mitfa* expression in the developing retina, suggesting that the Gal4-UAS elements were functioning correctly in the transgenic larvae (Fig. 9C,D). Cryosections of 5 dpf GFP positive embryos contained scattered patches of pigmented cells throughout the retinal layers in a pattern consistent with the observed GFP expression (Fig 9E–G). This experiment demonstrates that *mitfa* is capable of inducing pigmentation in the developing zebrafish eye *in vivo* and provides additional support for the notion that *mitfa* and *mitfb* may have a non-essential role in zebrafish RPE development.

## Discussion

In this study we have demonstrated that in zebrafish, Otx (Otx1a and Otx2, with some contribution from Otx1b) but not Mitf (Mitfa and Mitfb) transcription factors are required for normal RPE development, and that their absence causes a delay in gene expression and pigmentation culminating, in the most severe cases, in significant coloboma. While the role of Otx in eye formation therefore appears to be broadly conserved between zebrafish and other vertebrates [9], we found in contrast that the loss of Mitf transcription factors encoded by the duplicated loci *mitfa* and *mitfb* does not create RPE abnormalities in zebrafish, nor the microphthalmia phenotype for which the mouse locus was named.

This surprising result might be simply explained if the *mitfa* and/or *mitfb* mutants used in this study retain residual activity. We believe this is unlikely for several reasons. First, three of the four alleles in question (*mitfa^w2^*, *mitfb^hu3857^* and *mitfb^hu3561^*) create premature stop codons, and one of these (*hu3561*) lies within the helix-loop-helix (HLH) dimerization domain. The remaining allele, *mitfa^b692^*, is a missense mutation but is also located in the HLH domain, and besides being insufficient for development of neural crest melanocytes, shows no activity in a reporter assay [25]. The exons containing the stop codons in *w2* (exon 3) and *hu3857* (exon 2) could conceivably be skipped while preserving the reading frame, but we have been unable to detect expression of any such splice forms during embryogenesis. We have in fact identified a second isoform of *mitfa* with a novel first exon which splices into the third exon of the originally reported isoform, and a promoter capable of driving expression in the RPE (B.M.L. and J.A.L., unpublished results). However, it is predicted that both the *mitfa^w2^* and *mitfa^b692^* mutations would affect this isoform as well. Finally, regarding *mitfb* loss of function, we have tested several splice-blocking morpholinos against this gene and despite finding more than one that was highly effective as evaluated by RT/PCR, none produced an embryonic phenotype in wildtype or *mitfa* homozygotes (B.M.L. and J.A.L., unpublished results).

We have recently shown that another member of the MiT family, *tfec*, is also expressed in the developing zebrafish RPE [44]. Although it is apparently insufficient to compensate for loss if *Mitf* in mammals, *Tfec*, along with *Mitf*, was reported to be upregulated in the *ocular retardation* (*or*) mouse, in which neural retina is transfated to pigmented retina as a result of mutation of the *Chx10* gene [41]. Recently, *Tfec* was shown to rescue the small-eye phenotype of a strong mouse *Mitf* allele when misexpressed [45]. The zebrafish *tfec* gene is therefore a strong candidate for another factor that may compensate for the loss of *mitfa* and *mitfb*.

**Figure 10:**
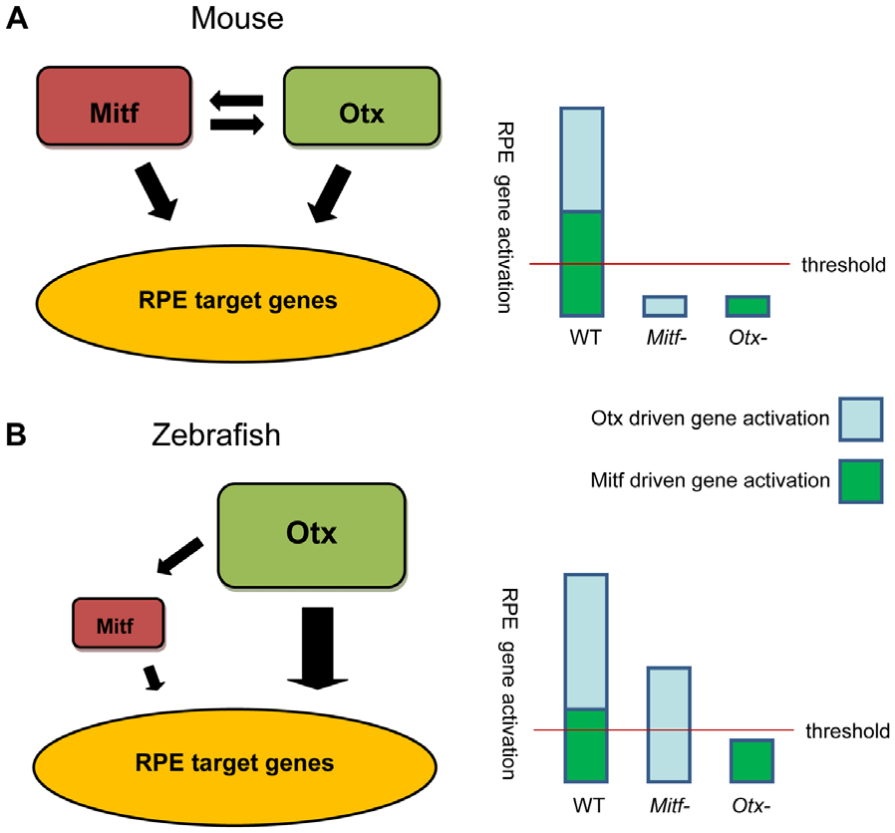
Models for transcriptional regulation of RPE development in mouse versus zebrafish. (A) In mouse, Otx and Mitf regulate each other's expression and that of other RPE target genes, such that loss of either Mitf or Otx results in insufficient activation to specify RPE cell fate. (B) In contrast, in zebrafish we postulate that Otx is a more important regulator of RPE target genes, and that Otx regulates Mitf expression but not the reverse. Correspondingly, loss of Otx activity results in sub-threshold RPE gene activation and loss of specification, but loss of Mitf alone does not. Loss of both genes produces even lower target gene activation and a more severe phenotype.

Another finding from these experiments is the lack of cross-regulation between zebrafish Otx and Mitf transcription factors. This data suggests a model in which a threshold level of gene activation, driven by Otx and Mitf together, is required for proper development of the RPE (Fig. 10). Under normal conditions, there is sufficient combined target gene activation by the two sets of transcription factors to adequately direct the development of the RPE. In mice, each factor is individually important, and the loss of either *Otx* or *Mitf* activity will lead to down-regulation of the other transcription factor [10], [12] (Fig. 10A–B). In zebrafish, Otx plays a more prominent role, and rather than a reciprocal relationship, Otx regulates Mitf but not vice-versa. Hence, the loss of the Mitf activity in *mitf* mutants leaves Otx activity unaffected with enough RPE target gene activation to surpass the threshold level required for RPE development, but when Otx activity is knocked down, *mitf* expression is also decreased, causing the level of RPE target gene expression to dip below the required threshold, leading to a varying degree of RPE abnormalities based on the severity of the decrease in this expression (Fig. 10C–D). This model of an RPE threshold is supported by the apparent variance in phenotype between eyes of a single animal that is observed in other species with *Mitf* and *Otx* mutations [12], [22]. If the level of RPE target gene activation is near the threshold of proper development, small changes may push the phenotype in either direction.

In addition to the differences between zebrafish and mice in the transcription factors required for RPE development, there are also differences in the ocular phenotype. Murine *Mitf* and *Otx* mutants experience the formation of a hyperproliferative, second inverted retinal layer in place of the RPE cells [12], [23]. Preliminary histology and analysis of double cone photoreceptors did not reveal any duplication of the retinal layers in zebrafish *otx* morphants. As the missing prospective RPE cells do not appear to transdifferentiate into retinal cells in zebrafish *otx* morphants, it is possible that they instead experience migration defects, fail to proliferate, or simply die off early in development. The eyes of most zebrafish *otx1a*/*otx2* morphants also do not show any reduction in size, and the retinal layers remain intact and organized in the unaffected dorsal regions of the eye. Previous evidence exists in zebrafish for the orderly lamination of the retina in the absence of RPE [46]. The morpholino paradigm used in our experiments has been reported to deplete embryos of detectable Otx2 during late somitogenesis stages [20], but we cannot definitively say to what degree the potential recovery of Otx expression may mitigate later phenotypes. Nor, for that matter, can we rule out a role for Otx protein expressed at earlier stages [20].

In spite of the differences we have identified in zebrafish compared to other experimental models, these experiments provide a better understanding of the regulatory pathways that may also control human RPE development. Similar to what has been observed in zebrafish, mutations in *OTX2* but not *MITF* have been identified in cases of severe human RPE developmental abnormalities such as microphthalmia and anophthalmia [15]. The phenotypic variance observed between the eyes of zebrafish is similar to what is observed in human patients with *OTX2* mutations. Finally, it appears that zebrafish may be similar to humans in the lack of dependence on *Mitf* for proper eye morphogenesis, as in contrast to mice the most severe human *MITF* mutations result in Tietz syndrome, a condition which involves relatively minor eye abnormalities relative to the effect on neural crest melanocytes [47].

## Supporting Information

Figure S1 **Otx knockdown does not affect lamination or dorsal eye size.** Histograms showing measurements of the RPE (A), Outer nuclear layer (B) and the length of the dorsal half of the eye (C), comparing *otx1a*/*otx2* morphants and control (uninjected) larvae at 5 dpf. Equivalent sections from 15 different eyes were averaged and no significant differences were observed between the two groups (P>0.05).
(TIF)

Figure S2 **Otx knockdown leads to a decrease of key RPE related genes.** (A–F) Expression of *silvb* was examined in *otx* morphants at 24 hpf and representative images are shown. At 24 hpf, a small percentage of the single *otx1a* (B), *otx1b* (C) and *otx2* (D) morphants displayed a slight loss of *silvb* expression in the ventral eye when compared to controls (A). Combined *otx1a*/*otx2* morphants showed a more severe reduction (E) and expression was almost completely eliminated in *otx1a*/*otx1b*/*otx2* morphants (F).
(TIF)

Figure S3 **No changes to the RPE and retinal layers are observed in zebrafish** ***Mitf*** **mutants.** (A) Equivalent sagittal sections from at least seven individual specimens for each genotype were analyzed using ImageJ software. RPE and outer nuclear layer thickness were measured at the central retina and eye length was measured at the proximal point of the lens. ANOVA analysis revealed no significant differences in RPE thickness (A, P = 0.115), ONL thickness (B, P = 0.156), or total eye length (C, P = 0.069).
(TIF)

Figure S4 **Results of individual trials for** ***otx1a/otx2*** **knockdown in** ***mitf*** **mutants.** The results from *otx1a/otx2* knockdown in *mitf* mutants were too variable to interpret as a single group but all demonstrate a significant difference between phenotypic outcomes in *mitfa;mitfb* double mutants to wildtype and single *mitf* mutants. The phenotypic variability in phenotype-to-genotype correlations between trials is likely the result of the potency of the morpholino combination and the difficulty in replicating exact injection conditions between trials. On the left are tabulated the raw eye phenotype data for the three additional trials and on the right are displayed the same data as phenotype percentages for each genotype. The *mitfa;mitfb* double mutants displayed a significantly higher percentage of eyes with major phenotypes when compared to wildtype and single *mitf* mutants in all trials (p<0.0001).
(TIF)

Figure S5 **Single morpholino knockdown of** ***otx1a*** **or** ***otx2*** **in** ***mitf*** **mutants.** Injection of *otx1a* (A) or *otx2* (B) morpholinos individually at a concentration of 2 ng/embryo produced a significantly greater percentage (P<0.0001) of eye defects in *mitfb^hu3857^*;*mitfa^w2^* double mutants compared to injections of wildtype and single *mitf* mutants. (A) wildtype, N = 476; *mitfa*, N = 372; *mitfb*, N = 464; *mitfa*;*mitfb*, N = 472. (B) wildtype, N = 368; *mitfa*, N = 330; *mitfb*, N = 220; *mitfa*;*mitfb*, N = 274.
(TIF)
