# Supplementary figures and images for: Otx but Not Mitf Transcription Factors Are Required for Zebrafish Retinal Pigment Epithelium Development
Source: PLoS One. 2012 Nov 5;7(11):e49357. doi: 10.1371/journal.pone.0049357 (PMC3489725; doi:10.1371/journal.pone.0049357)

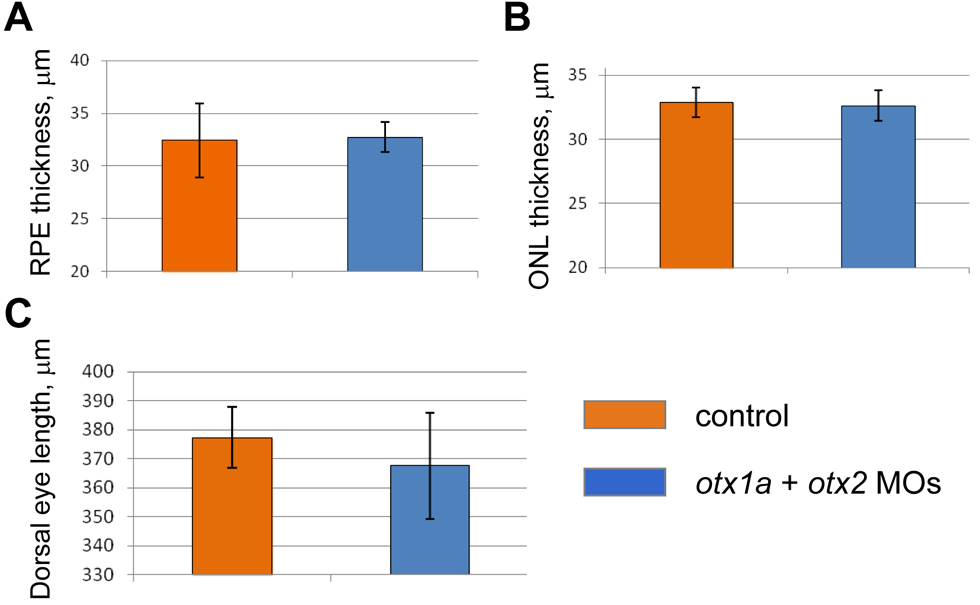

Supplement: Figure S1 — Otx knockdown does not affect lamination or dorsal eye size. Histograms showing measurements of the RPE (A), Outer nuclear layer (B) and the length of the dorsal half of the eye (C), comparing otx1a/otx2 morphants and control (uninjected) larvae at 5 dpf. Equivalent sections from 15 different eyes were averaged and no significant differences were observed between the two groups (P>0.05). (TIF) [file pone.0049357.s001.tif]

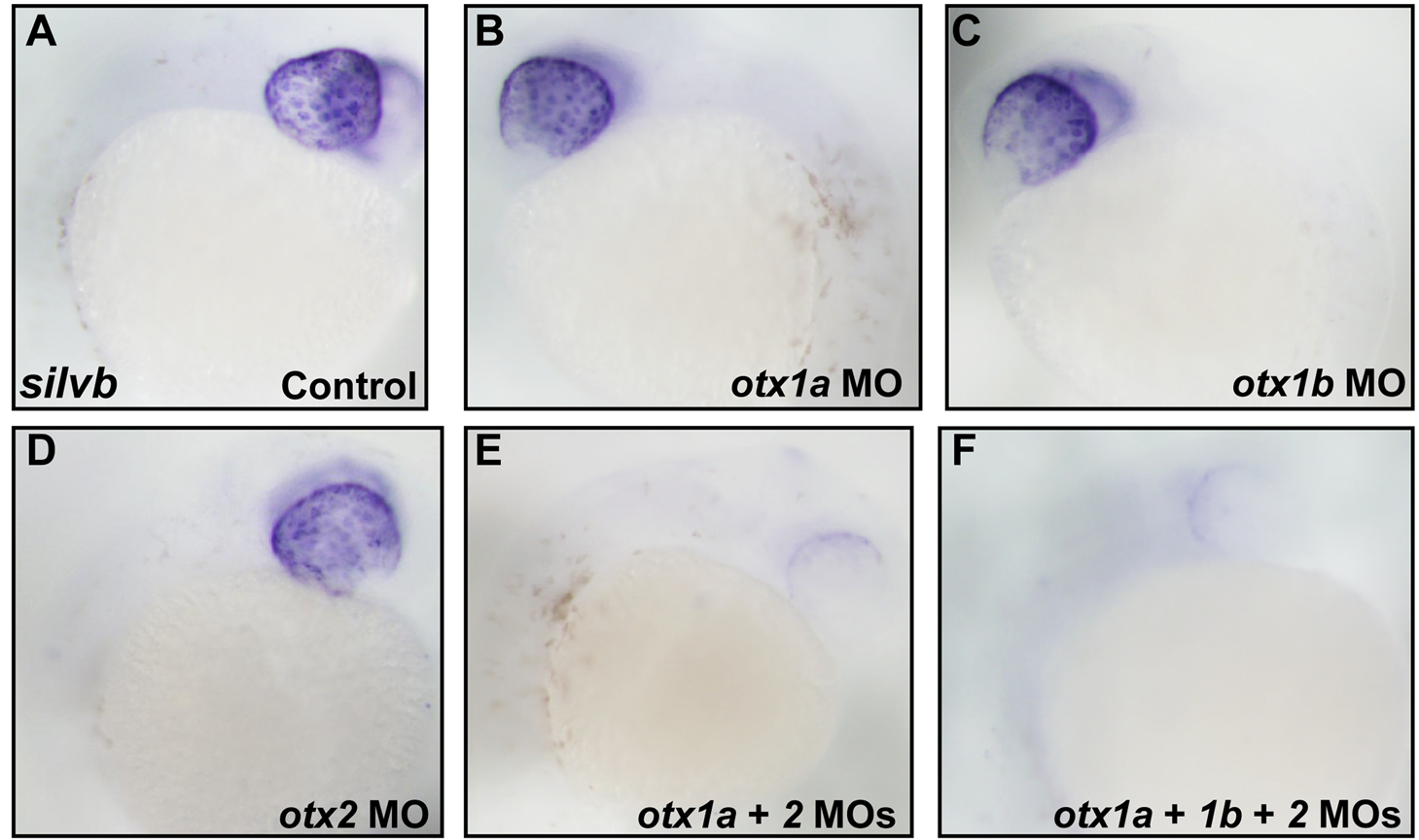

Supplement: Figure S2 — Otx knockdown leads to a decrease of key RPE related genes. (A–F) Expression of silvb was examined in otx morphants at 24 hpf and representative images are shown. At 24 hpf, a small percentage of the single otx1a (B), otx1b (C) and otx2 (D) morphants displayed a slight loss of silvb expression in the ventral eye when compared to controls (A). Combined otx1a/otx2 morphants showed a more severe reduction (E) and expression was almost completely eliminated in otx1a/otx1b/otx2 morphants (F). (TIF) [file pone.0049357.s002.tif]

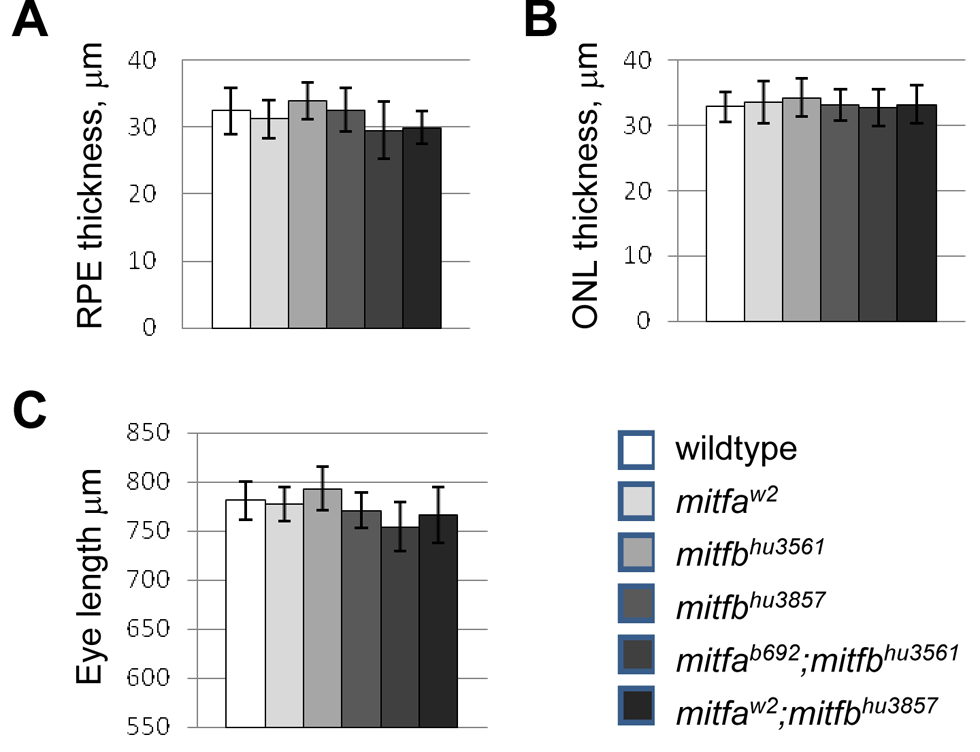

Supplement: Figure S3 — No changes to the RPE and retinal layers are observed in zebrafish Mitf mutants. (A) Equivalent sagittal sections from at least seven individual specimens for each genotype were analyzed using ImageJ software. RPE and outer nuclear layer thickness were measured at the central retina and eye length was measured at the proximal point of the lens. ANOVA analysis revealed no significant differences in RPE thickness (A, P = 0.115), ONL thickness (B, P = 0.156), or total eye length (C, P = 0.069). (TIF) [file pone.0049357.s003.tif]

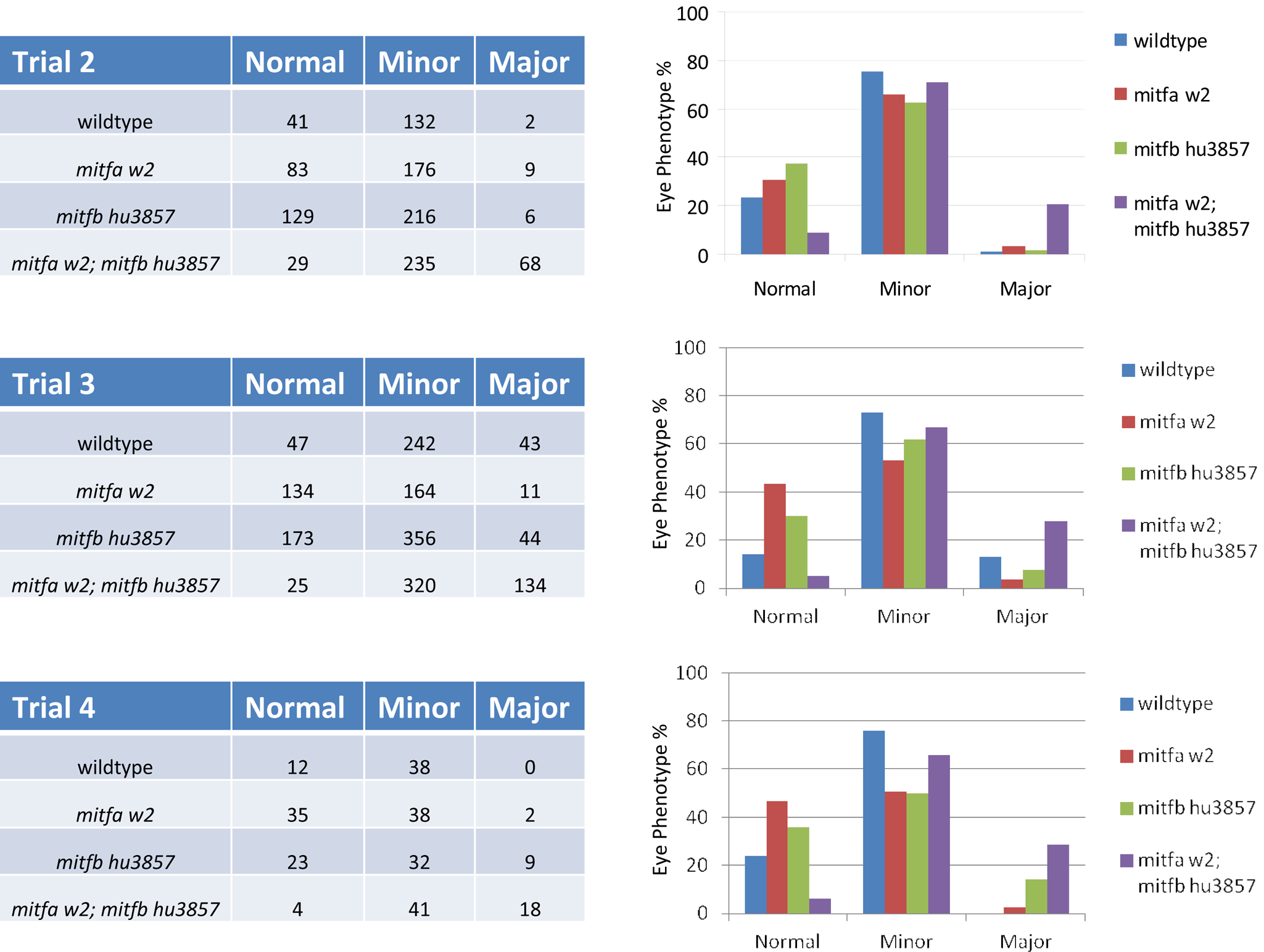

Supplement: Figure S4 — Results of individual trials for otx1a/otx2 knockdown in mitf mutants. The results from otx1a/otx2 knockdown in mitf mutants were too variable to interpret as a single group but all demonstrate a significant difference between phenotypic outcomes in mitfa;mitfb double mutants to wildtype and single mitf mutants. The phenotypic variability in phenotype-to-genotype correlations between trials is likely the result of the potency of the morpholino combination and the difficulty in replicating exact injection conditions between trials. On the left are tabulated the raw eye phenotype data for the three additional trials and on the right are displayed the same data as phenotype percentages for each genotype. The mitfa;mitfb double mutants displayed a significantly higher percentage of eyes with major phenotypes when compared to wildtype and single mitf mutants in all trials (p<0.0001). (TIF) [file pone.0049357.s004.tif]

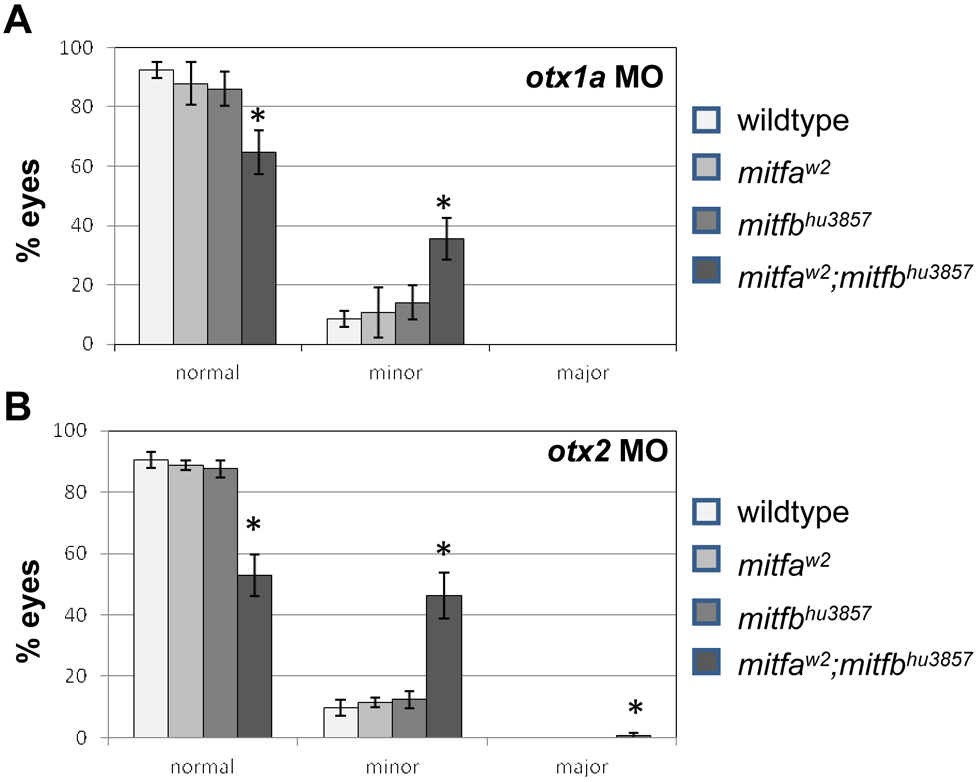

Supplement: Figure S5 — Single morpholino knockdown of otx1a or otx2 in mitf mutants. Injection of otx1a (A) or otx2 (B) morpholinos individually at a concentration of 2 ng/embryo produced a significantly greater percentage (P<0.0001) of eye defects in mitfbhu3857;mitfaw2 double mutants compared to injections of wildtype and single mitf mutants. (A) wildtype, N = 476; mitfa, N = 372; mitfb, N = 464; mitfa;mitfb, N = 472. (B) wildtype, N = 368; mitfa, N = 330; mitfb, N = 220; mitfa;mitfb, N = 274. (TIF) [file pone.0049357.s005.tif]
